# Loss of heterozygosity of *CDKN2A (p16INK4a)* and *RB1* tumor suppressor genes in testicular germ cell tumors

**DOI:** 10.2478/v10019-010-0035-7

**Published:** 2010-09-09

**Authors:** Tomislav Vladusic, Reno Hrascan, Nives Pecina-Slaus, Ivana Vrhovac, Marija Gamulin, Jasna Franekic, Bozo Kruslin

**Affiliations:** 1 Department of Biochemical Engineering, Faculty of Food Technology and Biotechnology, University of Zagreb, Zagreb, Croatia; 2 Laboratory of Neurooncology, Croatian Institute for Brain Research, Department of Biology, School of Medicine, University of Zagreb, Zagreb, Croatia; 3 Department of Oncology, Rebro University Hospital Center, Zagreb, Croatia; 4 Ljudevit Jurak Department of Pathology, Sisters of Mercy University Hospital, Zagreb, Croatia

**Keywords:** loss of heterozygosity, *CDKN2A*, *RB1*, seminomas, nonseminomas

## Abstract

**Background:**

Testicular germ cell tumors (TGCTs) are the most frequent malignances in young adult men. The two main histological forms, seminomas and nonseminomas, differ biologically and clinically. pRB protein and its immediate upstream regulator p16INK4a are involved in the RB pathway which is deregulated in most TGCTs. The objective of this study was to evaluate the occurrence of loss of heterozygosity (LOH) of the *CDKN2A (p16INK4a)* and *RB1* tumor suppressor genes in TGCTs.

**Materials and methods.:**

Forty TGCTs (18 seminomas and 22 nonseminomas) were analyzed by polymerase chain reaction using the restriction fragment length polymorphism or the nucleotide repeat polymorphism method.

**Results:**

LOH of the *CDKN2A* was found in two (6%) out of 34 (85%) informative cases of our total TGCT sample. The observed changes were assigned to two (11%) nonseminomas out of 18 (82%) informative samples. Furthermore, LOH of the *RB1* was detected in two (6%) out of 34 (85%) informative cases of our total TGCT sample. Once again, the observed changes were assigned to two (10.5%) nonseminomas out of 19 (86%) informative samples. Both LOHs of the *CDKN2A* were found in nonseminomas with a yolk sac tumor component, and both LOHs of the *RB1* were found in nonseminomas with an embryonal carcinoma component.

**Conclusions:**

The higher incidence of observed LOH in nonseminomas may provide a clue to their invasive behavior.

## Introduction

Testicular germ cell tumor (TGCT) is diagnosed mainly after puberty and is the most frequent malignancy in young adult men^1^, however, it is also not rare in childhood.^2^ The two main histological forms, seminomas and nonseminomas, differ biologically and clinically. About 50% of TGCTs are pure seminomas and 40% pure or mixed nonseminomas. The remaining 10% containing both seminoma and nonseminoma components are classified as being nonseminoma according to the World Health Organization (WHO) classification system.^3^ The genetic alterations underlying the development of these neoplasms have not been understood fully, although much has been done to elucidate them.^4^,^5^

The cell cycle regulatory pathway deregulated in almost all human tumors appears to be the G_1_ phase-controlling mechanism centered around the pRB protein. Different cancers seem to have altered different key components of that mechanism, which may be connected with gene activity patterns in different target cells.^6^ The mechanism involves pRB and its immediate upstream regulators, the cyclin dependent kinases (CDK4 and CDK6), their catalytic partners (cyclin D1, cyclin D2 and cyclin D3), and the members of the INK4 family of CDK inhibitors (p16INK4a, p15INK4b, p18INK4c and p19INK4d). This mechanism seems to be a common point for various signaling pathways, serving as a growth factor dependent cell cycle switch. Deregulation of the RB pathway may be an obligatory step in oncogenesis, making tumor cells less dependent on growth stimuli.^6^,^7^

The pRB is essential in cell cycle regulation and its function is regulated by phosphorylation. In G_0_ and the early G_1_ phase, hypophosphorylated pRB is complexed with the transcription factor E2F.^8^ In late G_1_, a significant hyperphosphorylation of the pRB by CDK4 and CDK6 in complex with D cyclins (D1, D2, D3) occurs.^9^

The *CDKN2* locus at chromosomal region 9p21 encodes p16INK4a tumor suppressor protein involved in the RB cell cycle control pathway.^10^ p16INK4a functions as a regulator of G_1_/S phase transition by inhibiting the activity of CDK4 and CDK6. Thus, by inhibiting pRB phosphorylation, p16INK4a can promote the formation of a pRB-E2F repressive transcriptional complex, which blocks cell cycle progression past G_1_/S restriction point.^11^

In diverse types of cancer the RB pathway becomes deregulated through alterations in one or more of its components. The most common defects of the RB pathway are mutations or deletions of *RB1* and inactivating mutations or promoter methylation of the *CDKN2A* (*p16INK4a*) tumor suppressor gene, as well as the overexpression of the cyclin D2/CDK4 complex.^6^,^12^,^13^

The objective of this study was to evaluate the occurrence of the loss of heterozygosity (LOH) of the *CDKN2A* and *RB1* tumor suppressor genes in TGCTs.

## Materials and methods

### Patients and tumor material

Fourty TGCT samples (18 seminomas and 22 nonseminomas) were collected from Sisters of Mercy University Hospital and University Hospital Center, Zagreb, Croatia. The samples were formalin-fixed and paraffin-embedded. Clinical and pathological data for 40 TGCTs according to the WHO 2004 classification are shown in Table 1.

### DNA extraction

For each specimen, 20 μm paraffin-embedded section was prepared for DNA extraction. In addition, 4 μm section was stained with hematoxylin-eosin to identify the tumor and normal tissue areas which were removed separately from the microscopic slide, transferred to microtubes and extracted using QIAamp DNA Mini Kit (Qiagen, Hilden, Germany).

### LOH analysis of CDKN2A gene

A previously described polymorphic microsatellite marker hMp16α-I1 consisting of a mononucleotide tract of (A)_23_ located close to intron 1 of the *CDKN2A* gene was analyzed in this study.^14^ Primers used for polymerase chain reaction (PCR) amplifications were 5’-CAATTACCACATTCTGCGCTT-3’ and 5’-CAGGCAGAGAGCACTGTGAG-3’, which produced 190–210 bp fragments. PCR amplifications were performed in 25 μl reaction volume with a final concentration 0.2 mM of each dNTP, 3 mM MgCl_2_, 0.2 μM of each primer (Sigma-Aldrich, St. Louis, MI, USA), 1x Flexi buffer (Promega, Madison, WI, USA) and 0.5 U of GoTaq^®^ Hot Start Polymerase (Promega, Madison, WI, USA). One hundred nanograms of DNA were used in each PCR reaction. PCR amplifications were carried out in a Eppendorf Mastercycler Personal (Hamburg, Germany), with cycling times of 96ºC for 5 min (one cycle), then 45 cycles of 96ºC for 30 s, 57ºC for 45 s, and 72ºC for 30 + 1 s. The final step was incubation at 72º C for 10 min. Amplified DNA fragments were analyzed on silver-stained 15% polyacrylamide gels. LOH of *CDKN2A* was considered to had occured if one out of two alleles (heterozygous samples) of a gene marker was missing or significantly reduced in comparison to alleles from adjacent normal tissue.

### LOH analysis of RB1 gene

LOH of *RB1* was detected using polymerase chain reaction-restriction fragment length polymorphism method (PCR-RFLP). Amplification with *RB1* primers 5′-TCCCACCTCAGCCTCCTTAG-3’ and 5′-GTAGGCCAAGAGTGGCAGCT-3′ used in our study produced a 190 bp segment of intron 17.^15^ PCR amplifications were performed under conditions mentioned above. To generate the RFLP pattern for LOH analysis, 10 μl of PCR product were digested with 5 U of XbaI restriction enzyme (Fermentas, Vilnius, Lithuania) in a total volume of 25 μl for 12 h. The restriction digestion resulted in fragments of 75 and 115 bp. DNA fragments were analyzed on silver-stained 15% polyacrylamide gels. LOH was recognized as a partial or complete loss of either the uncleaved (190 bp) or the cleaved (75 + 115 bp) allele.

## Results

**Figure 1:**
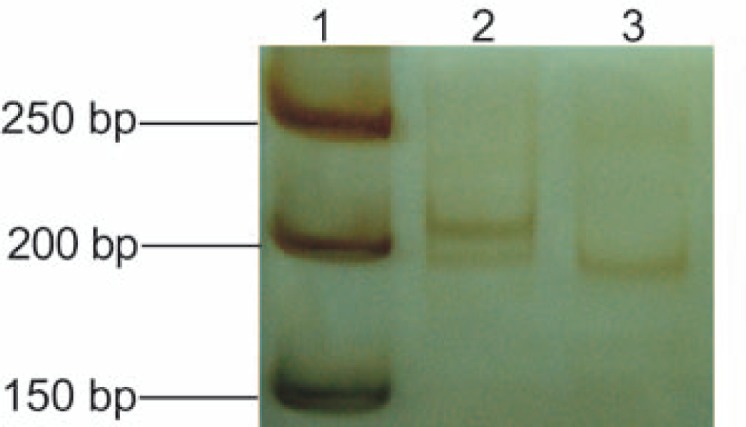
Loss of heterozygosity (LOH) of the *CDKN2A* gene at polymorphic microsatellite marker hMp16α-I1. Silver-stained 15% polyacrylamide gel. Lane 1: 50-bp DNA ladder (Fermentas, Vilnius, Lithuania); lane 2: heterozygous normal testis tissue; lane 3: LOH in the corresponding testicular germ cell tumor (nonseminoma, patient no. 31).

In this study 40 TGCTs, 18 seminomas and 22 nonseminomas, were analyzed. First, we searched for LOH of the intragenic polymorphic microsatellite marker hMp16α-I1 in the *CDKN2A* gene. From 40 TGCTs, 34 (85%) tumors were informative for this polymorphism, 16 (89%) seminomas and 18 (82%) nonseminomas. Our analysis revealed that two (6%) samples showed LOH of hMp16α-I1 marker. The observed changes were assigned to two nonseminomas (11%, patients no. 31 and 34, Table 2). In both tumor cases, one out of two allels of gene marker was missing in comparison to alleles from the adjacent normal tissue (Figure 1). In addition, both LOHs of the *CDKN2A* were found in nonseminomas with a yolk sac tumor component. LOH of the *CDKN2A* gene was not observed among seminomas.

**Figure 2:**
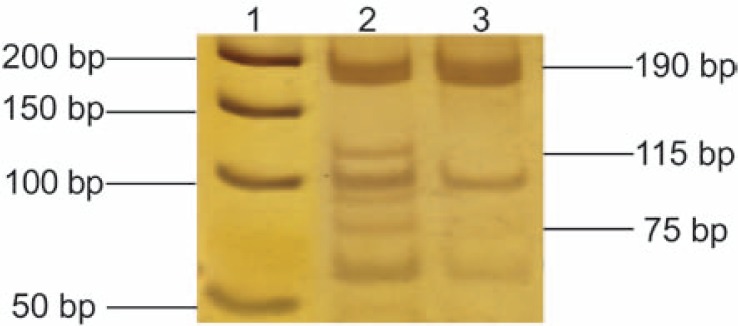
Loss of heterozygosity (LOH) of the *RB1* gene at intron 17 (XbaI restriction polymorphism). Silver-stained 15% polyacrylamide gel. Lane 1: 50-bp DNA ladder (Fermentas, Vilnius, Lithuania); lane 2: heterozygous normal testis tissue; lane 3: LOH in the corresponding testicular germ cell tumor (nonseminoma, patient no. 25).

The analysis of intragenic polymorphic restriction marker of the *RB1* gene showed that 34 (85%) of total TGCTs were heterozygous for this polymorphism; 15 (83%) seminomas and 19 (86%) nonseminomas. LOH was observed in two (6%) samples when looking at the total TGCTs analyzed. Once again the observed allelic losses were assigned to nonseminomas: two samples (10.5%, patients no. 20 and 25, Table 2) had one of the alleles missing in comparison to bands from the adjacent normal testis tissue. These nonseminoma samples showed loss of the cleaved allele (75- and 115-bp fragments), as the single uncleaved allele (190-bp fragment) appeared on the silver stained 15% polyacrylamide gel (Figure 2). Furthermore, both LOHs of the *RB1* were found in nonseminomas with an embryonal carcinoma component. None of the seminomas demonstrated LOH of the *RB1* gene.

No statistically relevant correlation between the occurrence of LOH, form of TGCT, histological type of contained components and tumor stage according to TNM classification could be determined by Fisher’s exact test.

## Discussion

TGCT is associated with characteristic abnormalities in the RB pathway including upregulation of cyclin D2, and downregulation of pRB and the CDK inhibitors such as p16INK4a.^7^

The inactivation of the *CDKN2A* gene, which encodes an inhibitor of CDK4 and CDK6, is one of the most common molecular events in human neoplasms. The major mechanisms contributing to *CDKN2A* silencing are promoter methylation, gene mutations and hemizygous or homozygous deletions. When one *CDKN2A* allele is mutated or methylated, the second allele is often deleted.^16^

The analysis of the expression of INK4 family has pointed to a down-regulation of *CDKN2A* in testicular neoplasms.^7^,^12^ Honorio *et al*.^17^ demonstrated that promoter hypermethylation of that gene is not involved in the decrease of p16INK4a protein expression. In contrast, some studies have found promoter mutation, a half of analyzed TGCTs had *de novo* promoter methylation and approximately half of TGCTs showed hypermethylation of *CDKN2A* exon 1α. All that correlated with a decreased level of *CDKN2A* mRNA expression.^1^,^18^ However, Chaubert *et al*.^18^ have not detected any *CDKN2A* mutations and observed LOH of the *CDKN2A* gene in only one of 29 TGCTs with a yolk sac tumor component, using seven different markers. These observations indicate that *CDKN2A* gene inactivation might be an important mechanism leading to cell deregulation in TGCTs.

Despite of promoter methylation and mutations being the most common ways of inactivating *CDKN2A* in TGCTs, various studies detected LOH at the position of the *CDKN2A* gene, varying from as low as 5.5% to as high as 42%. The LOHs of *CDKN2A* were reported mostly in nonseminomas.^5^,^19^ Genomic region containing *CDKN2A* (9p21) is reported to be the most commonly deleted region early in the development of nonseminomas, which may be implicated in their ability to differentiate into various types, for various markers located within this region.^20^

In our study only nonseminomas demonstrated LOH (Table 2). Both LOHs of the *CDKN2A* were found in nonseminomas with a yolk sac tumor component, one sample also having an embryonal carcinoma component. Furthermore, one nonseminoma with the LOH of *CDKN2A* demonstrated LOH of *TP53* gene, and the other showed LOH of the *CDH1* gene.^21^

The *RB1* gene is often deleted or mutated to an inactive form in a variety of human tumors. Cells of embryonal testes and intratubular germ cell neoplasia (ITGCN) show no expression of pRB, whereas it is expressed in healthy testes during spermatogenesis. The lack of pRB in most TGCTs may, therefore, reflect its deregulation by normal mechanisms in testicular germ cells. However, the lack of pRB may facilitate the transition of those cells to tumor cells of ITGCN and thus contribute to molecular pathogenesis of TGCTs.^7^,^12^ Lowered levels of pRB mRNA compared with normal testis did not reflect a grossly altered structure of the DNA coding regions, but instead relates to a potentially reversible transcriptional modulation through the promoter methylation. The pRB appears to be differentially expressed according to the differentiation status of the tumor, more differentiated cells of teratocarcinoma show positive immunohistochemical staining, less differentiated forms of TGCT such as embryonal carcinoma are stained negatively.^12^,^22^,^23^

In contrast, deletions of *RB1* gene are, along with its mutations, also reported as one of the most common alterations of the RB pathway. Various studies revealed deletions of the *RB1* gene region in testicular cancer.^5^ For example, Peng *et al*.^24^ used short variable number of tandem repeats in *RB1* introns 16 and 20, and found LOH in 5% of seminomas and 28% of nonseminomas analyzed within 93% of informative TGCT cases. The location of the *RB1* gene is reported to be one of the most commonly involved in allelic imbalance within TGCTs.^4^ The exact alterations of the *RB1* in various forms of TGCTs needs to be further elucidated in more detail. Studies also revealed a different pattern of LOH in different histological types of nonseminomas for markers located within the genomic region containing the *RB1* gene (13q14), varying from 0% in yolk sac tumor component to 50% in choriocarcinoma.^25^

In our study, LOH of the *RB1* gene was found in nonseminomas with an embryonal carcinoma component, and both nonseminomas with LOH of *RB1* also demonstrated LOH of the *TP53* gene.^21^ Interestingly, the amount of embryonal carcinoma component in TGCT, along with vascular invasion, has been proved so far to be the only clinically valid prognostic factor for the development of stage II metastatic testicular cancer.^26^

LOH of *CDKN2A*, *RB1*, *TP53* and *CDH1* in TGCTs may increase their tumorigenic potential by the increased proliferation capacity due to *RB1* loss and decreased rate of apoptosis due to *TP53* alteration.^19^,^21^,^27^ It has been shown that TP53 is abundant but inactive in cells of TGCTs. In healthy testes such reversibly inactivated TP53 may play a role in switching between proliferation and apoptosis in cells undergone meiosis.^27^ It was reported that, in cells that sustained lesion in the RB pathway, there was a strong selection for the loss or inactivation of wild type TP53. Alterations of *RB1* are often seen together with alterations of *TP53* in variety of different cancers.^6^,^10^,^15^ It is possible that the inactivation of both *RB1* and *TP53* genes in a cell produces a synergistic effect, which imposes a stronger selective pressure for the cellular transformation. This may also help to explain the high proliferation rate and/or invasiveness of TGCTs with embryonal carcinoma and yolk sac tumor component. A higher incidence of LOH in nonseminomas may provide a clue to their invasive behavior, because for some of the nonseminoma types there seem to be a region of preferential loss (3q27–3q28 in embryonal carcinoma), and all of the TGCTs show gain of 12p11–12p12 sequences.^20^ Knowing the exact nature of genetic alterations associated with these tumors may provide novel treatment strategies.^28^

However, the low frequency of observed LOHs in this study could be a consequence of genomic instability in above mentioned nonseminomas, rather than the main cause of *CDKN2A* and *RB1* inactivation.^24^

## Figures and Tables

**TABLE 1:** Clinical and pathological data for 40 testicular germ cell tumor cases

Patient no.*	**Age**	**pTNM**	**Histology**
**1**	26	pT1NXMX	ITGCN, S
**2**	26	pT1NXMX	ITGCN, S
**3**	37	pT1NXMX	S
**4**	33	pT1NXMX	ITGCN, S
**5**	31	pT1NXMX	ITGCN, S
**6**	29	pT1NXMX	ITGCN, S
**7**	39	pT1NXMX	ITGCN, S
**8**	27	pT3NXMX	S
**9**	41	pT1NXMX	ITGCN, S
**10**	48	pT1NXMX	S
**11**	48	pT2NXMX	S
**12**	34	pT1NXMX	ITGCN, S
**13**	60	pT1NXMX	ITGCN, S
**14**	29	pT1NXMX	ITGCN, S
**15**	60	pT1NXMX	S
**16**	29	pT1NXMX	ITGCN, S
**17**	28	pT1NXMX	ITGCN, S
**18**	32	pT1NXMX	ITGCN, S
**19**	37	pT1NXMX	EC
**20**	18	pT2NXMX	EC, IT, MT, S
**21**	24	pT1NXMX	EC, ITGCN, S
**22**	22	pT2NXMX	EC, YST
**23**	37	pT1NXMX	EC, ITGCN, S
**24**	28	pT2NXMX	C, EC, IT, MT
**25**	17	pT2NXMX	EC, MT
**26**	34	pT2NXMX	EC
**27**	19	pT1NXMX	EC, ITGCN, MT, YST
**28**	39	pT1NXMX	MT, YST
**29**	21	pT2NXMX	EC, MT, YST
**30**	23	pT2NXMX	EC, IT, MT
**31**	22	pT1NXMX	MT, YST
**32**	25	pT3NXMX	EC
**33**	45	pT2NXMX	EC, ITGCN, S, YST
**34**	NK	pT2NXMX	C, EC, ITGCN, S, YST
**35**	23	pT2NXMX	EC, IT, ITGCN, MT, YST
**36**	39	pT1NXMX	EC, ITGCN, S, YST
**37**	24	pT2NXMX	EC, ITGCN, YST
**38**	30	pT1NXMX	EC, ITGCN, YST
**39**	36	pT1NXMX	EC, ITGCN, MT, YST
**40**	58	pT2NXMX	EC, ITGCN, YST

* seminomas, patients no. 1–18; nonseminomas, pateints no. 19–40

C = choriocarcinoma; EC = embryonal carcinoma; IT = immature teratoma; ITGCN = intratubular germ cell neoplasia; MT = mature teratoma; S = seminoma; YST = yolk sac tumor; NK = not known

**TABLE 2:** A) Observed loss of heterozygosity (LOH) and B) distribution of observed LOH of *CDKN2A* and *RB1* genes in testicular germ cell tumors

**A) observed LOH**		
**Patient no.**	**CDKN2A**	**RB1**

**20**		LOH
**25**		LOH
**31**	LOH	NI
**34**	LOH	I
**B) distribution of observed LOH**		
**Tumor**	**CDKN2A**	**RB1**

**Seminoma, Σ 18**	0% (0/16)	0% (0/15)
**Nonseminoma, Σ 22**	11% (2/18)	10.5% (2/19)

I = informative (heterozygous); NI = not informative (homozygous)

Numbers in parentheses: the number of tumors demonstrating LOH over the number of informative tumors.
